# Discriminatory Value and Validation of a Risk Prediction Model Based on Serum Cytokines in Pediatric Acute Appendicitis: A Single-Center Experience of 483 Cases

**DOI:** 10.3390/children12030298

**Published:** 2025-02-27

**Authors:** Jiajia Zhou, Guobin Liu, Xiaofeng Song, Quan Kang

**Affiliations:** Department of General Surgery and Trauma Surgery, Children’s Hospital of Chongqing Medical University, National Clinical Research Center for Child Health and Disorders, Ministry of Education Key Laboratory of Child Development and Disorders, China International Science and Technology Cooperation Base of Child Development and Critical Disorders, Chongqing Key Laboratory of Pediatric Metabolism and Inflammatory Diseases, Chongqing 400014, China

**Keywords:** appendicitis, cytokines, children

## Abstract

**Objectives**: Pediatric acute appendicitis (AA) is one of the most prevalent acute abdominal conditions in pediatric surgery. Children with complicated acute appendicitis (CA) may need timely surgical decisions and have a worse prognosis. In this study, we explored the risk factors and developed a predictive model for complicated AA in children. **Methods**: A retrospective analysis was conducted on patients data from those hospitalized for acute appendicitis, confirmed by post-surgery pathological results, at Children’s Hospital of Chongqing Medical University between September 2022 and October 2023. Lasso regression was performed to identify risk factors, and multivariate logistic regression analysis was used for model establishment. **Results**: Serum levels of IFN-γ, IL-5, IL-6, IL-8, and IL-10 before surgery were useful in classifying acute appendicitis in children. IL-6, IL-8, and IL-10, on their own, had high predictive values for CA in children. Independent risk factors for CA were age, IL-10, and IFN-γ. A multifactorial logistic regression prediction model was established, demonstrating good predictive efficacy. Its predictive sensitivity was 70.0%, specificity 73.9%, with an AUC of 0.7949. Furthermore, the results of the external validation indicated that the model’s accuracy was good, with an AUC of 0.8567. **Conclusions**: Early identification of CA is imperative for timely clinical decision-making. Prediction models based on age, IL-10, and IFN-γ may be reliable and accurate in predicting the incidence of CA, which may lead to better clinical outcomes for children with AA.

## 1. Introduction

Pediatric acute appendicitis (AA) is one of the most common acute abdominal conditions in pediatric surgery. According to a meta-analysis, 1–8% of children who arrive at the emergency room complaining of acute abdominal pain are ultimately found to have acute appendicitis [1]. Presently, the clinical diagnosis of AA is frequently based on a history of abdominal pain and symptoms of fixed pressure in the right lower abdomen. Abdominal ultrasonography and blood tests are frequently utilized as supplementary testing to aid in the diagnosis. Pediatric acute appendicitis is characterized by its quick onset, swift progression, and ease of perforation. If prompt and efficient treatment is not provided, the child’s condition may worsen, leading to poor prognosis. In extreme circumstances, it may even become life-threatening [2,3]. It was once thought that children with AA should have their appendices removed as soon as possible. Nonetheless, some academics have proposed recently that distinct treatment protocols could be created based on different kinds of pediatric AA [4]. Children with uncomplicated appendicitis may benefit from conservative treatment rather than surgical intervention [5,6]. On the other hand, children with complicated appendicitis may need early surgical intervention due to their considerably worse prognosis, higher treatment costs [7], six-fold higher readmission rate, and longer duration of hospital stay [8]. This implies that it is critical for doctors to distinguish between uncomplicated and complicated pediatric AA promptly and to provide the right care. However, this remains highly difficult because there is no universally agreed categorization, and clinical diagnostics, biomarkers, imaging, and scoring systems have not proven to be successful enough in distinguishing between pediatric uncomplicated and complicated appendicitis [9].

Serum cytokines are crucial mediators of the immunological and inflammatory responses of the body, and recent studies have extensively investigated and documented the association between appendicitis and cytokines. Many studies have demonstrated that cytokine levels can serve as predictive markers for assessing the course of acute appendicitis (AA), aiding in patient classification, diagnosis, staging, and prognosis. IL-6, IL-10, TNF-α, PCT, and CRP are among the most extensively researched ones. However, most previous studies using cytokines to assess the diagnosis and staging of appendicitis have only focused on one particular biomarker at a time [10], with many conducted solely on adult cohorts. In contrast, using a large retrospective cohort in this study, we examined the diagnostic performance of twelve serum cytokines between complicated and uncomplicated appendicitis in children, including IL-1, IL-2, IL-4, IL-5, IL-6, IL-8, IL-10, IL-12, IL-17, TNF-α, IFN-α, and IFN-β. We also developed a clinical prediction model based on independent risk factors among these cytokines in an attempt to provide a novel perspective on the diagnosis and management of acute appendicitis in pediatric patients.

## 2. Methods

### 2.1. Patients

**Figure 1:**
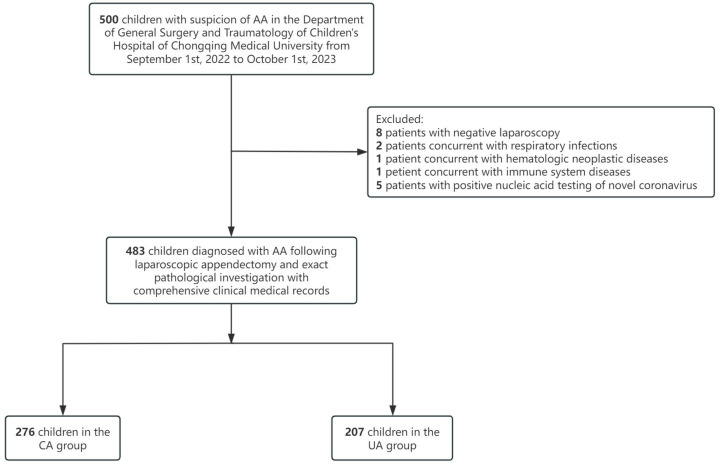
Study flowchart.

A total of 483 children diagnosed with AA in the Department of General Surgery and Traumatology of Children’s Hospital of Chongqing Medical University from 1 September 2022 to 1 October 2023 were recruited in this study. Patients with affirmed localized McBurney point tenderness, with or without positive ultrasound outcomes were considered as suspect AA. Inclusion criteria were as follows: (1) children diagnosed with AA following laparoscopic appendectomy and exact pathological investigation; (2) comprehensive clinical medical records. Exclusion criteria: (1) children with suspicion of AA undergoing laparoscopy but found not to have inflamed appendices; (2) concurrent with respiratory and urinary tract infections and other infectious disorders, inflammatory diseases; (3) concurrent with hematologic neoplastic diseases; (4) concurrent with immune system diseases; (5) positive nucleic acid testing of novel coronavirus. The study design is shown in Figure 1. This study was approved by the Ethics Committee of the Children’s Hospital of Chongqing Medical University (approval number: 98/2023).

### 2.2. Data Collection and Definitions

Clinical information was gathered retrospectively, which included age, sex, length of abdominal pain, clinical symptoms and signs (fever, vomiting, and diarrhea), hospital length of stay (LOS), complications (intestinal adhesions, intestinal obstruction, and sepsis), use of carbapenem antibiotics (imipenem and meropenem), and whether the postoperative period was transferred to the PICU. The results of twelve preoperative serum cytokine levels, including IL-1, IL-2, IL-4, IL-5, IL-6, IL-8, IL-10, IL-12, IL-17, TNF-α, IFN-α, and IFN-β, were documented, and postoperative histopathologic results were recorded. It is important to note that conducting such an investigation is a routine practice within our department. As part of our preoperative assessment protocol, cytokines level measurement is systematically performed to aid in the formulation of more precise treatment plans. Children with remarkably increased cytokines may undergo an appendectomy within 2 h of admission to the department. For such patients, when communicating the risks with the family, special emphasis will be placed on the severity of inflammation, the potential for significant adhesions during surgery, the possibility of a prolonged hospital stay, the potential need for carbapenem antibiotics, and the risk of postoperative complications such as intestinal obstruction and adhesions.

A total of 483 children were divided into groups based on the severity of intraoperative lesions and postoperative pathological findings from their appendectomies. In cases where there was a discrepancy between intraoperative findings and pathological results, the more severe result prevailed. This means that if either the intraoperative findings or the pathological results indicated CA, the patient was categorized into the CA group.

According to the 2020 WSES Jerusalem guidelines [11], the complicated appendicitis group (Complicated Appendicitis, CA) was defined by intraoperative findings and pathological results showing extensive tissue necrosis or perforation of the muscular layer of the appendix, suggestive of gangrenous and perforated appendicitis. Whereas, the presence of edema, congestion, or inflammatory infiltration of the appendix without necrosis or rupture were classified as uncomplicated appendicitis (Uncomplicated Appendicitis, UA).

### 2.3. Statistical Analysis

Continuous variables were expressed as the mean ± standard deviation (SD) or median with interquartile range [M(P, Ps)] and were analyzed by independent-sample *t*-tests, analysis of variance, and Mann–Whitney U tests for comparisons after checking the normality of the distribution with the Kolmogorov–Smirnov test. Categorical variables were expressed as numbers or percentages and were analyzed with the Chi-squared test or Fisher’s exact test. Lasso regression was performed to identify independent risk factors. Logistic regression results are shown with *p* values and odds ratios (ORs) with 95% confidence intervals (95% CIs), and a calculation formula for predicting probability was constructed with these independent risk factors. Stepwise regression was used to avoid interactions and multi-collinearity. SPSS version 25.0 (IBM Corporation, Armonk, NY, USA) and R 4.3.1 were used for statistical analysis. The area under the receiver operating characteristic (ROC) curve (AUC) was analyzed to assess the accuracy and to identify the cutoff point. A two-tailed *p* value < 0.05 was considered statistically significant.

Model validation included internal and external validation. Bootstrap sampling with 1000 replicates was utilized for internal validation. For external validation, 50 children with AA from Chongqing Medical University Children’s Hospital were included. The accuracy of the predictive model was assessed by substituting the prognostic data.

## 3. Results

### 3.1. Demographics and Clinical Manifestations at Baseline

A total of 483 children with acute appendicitis were included in this study, with 276 children in the CA group and 207 children in the UA group. The results of the comparison of the basic data and clinical characteristics of the two groups are shown in Table 1. Sex differences between the two groups were not statistically significant (*p* = 0.830). Age differences were statistically significant (*p* < 0.001) between the two groups, with the CA group having a younger age of onset than the CA group. There were statistically significant differences (*p* < 0.001) in the duration of abdominal pain, presence or absence of fever, and presence or absence of vomiting between the two groups. The CA group had a higher proportion of children experiencing fever and vomiting, and the duration of abdominal pain was relatively longer in this group. Due to the strong correlation between age and cytokine levels (Supplementary Table S1), we divided all patients into two age subgroups based on the median age to minimize the influence of age on subsequent model construction. In the subsequent analysis, age will be treated as a binary categorical variable (age group).

### 3.2. Preoperative Serum Cytokine Levels and Principle Component Analysis

Table 2 shows the findings of the comparison of the preoperative levels of twelve cytokines between the two groups. The CA group had greater preoperative levels of IL-5, IL-6, IL-8, and IL-10 than the UA group, while the UA group had lower preoperative levels of IFN-γ. These differences were statistically significant (all *p* < 0.01). ROC curves were used to plot the AUC values of IL-5, IL-6, IL-8, IL-10, and IFN-γ for the prediction of CA (Supplementary Figure S1), with the AUC values of IL-5 and IFN-γ being less than 0.5, indicating low predictive values. Further calculations of the cut-off values, specificity, and sensitivity of IL-6, IL-8, and IL-10 revealed that the AUC value of IL-6 was 0.794, indicating a relatively high level of specificity and sensitivity, with a potential specificity of 81.6%. IL-8 had an AUC of 0.604, with good sensitivity (63.8%) but low specificity (55.1%). IL-10 had an AUC of 0.746, with a sensitivity of 64.1% and a specificity of 73.4%.

Principal Component Analysis (PCA) is a prominent multivariate statistical algorithmic approach for distinguishing components using the six key mathematical steps that convert vectors to matrices. PCA is useful for lowering the dimensionality of complicated datasets to a simpler, manageable state while also limiting information loss [12]. In this study, we used principal component analysis (PCA) to visualize the twelve cytokine distributions of the CA group and the UA group to initially observe the differences in cytokine distributions between the two groups and to prepare for variable screening. Additionally, we utilized PCA and correlation coefficient matrix to evaluate the multicollinearity of the cytokines. PCA revealed that the two groups had more significant variations in the distribution of IL-6, IL-8, and IL-10. Furthermore, multicollinearity was observed between certain cytokines (Supplementary Figure S2).

### 3.3. Therapy and Prognosis

In comparison to the UA group, the CA group experienced a longer hospital stay, a higher percentage of children prescribed carbapenem antibiotics, and a higher percentage of complications, and these differences were statistically significant (*p* < 0.001). Between the two groups, there was no discernible difference in the percentage of children admitted to the PICU post-surgery (Table 3). As shown in Table 4, patients who received carbapenem antibiotics had higher levels of IL-1β, IL-6, IL-8, and IL-10 (*p* < 0.001). Children transferred to the PICU after surgery had higher IL-6, IL-8, and IL-10 levels. Additionally, IL-1β, IL-6, IL-8, IL-10, IFN-α, and IFN-γ levels were higher in children who developed complications.

### 3.4. Risk Factors, Establishment, and Validation of the Predictive Model for Pediatric CA

As the duration of abdominal discomfort, presence of fever, and presence of vomiting were more subjective, the above factors were not tested. LASSO regression was used to screen for factors in the remaining 13 independent variables (age and twelve cytokines) (Supplementary Figure S3a). The entire dataset was randomly partitioned into training and test sets in the ratio of 433:50, with the last 50 observations serving as the test set and the first 433 observations serving as the training set for modeling. Ten-fold cross validation was used to confirm the test model’s truthfulness. Age group, IL-5, IL-6, IL-8, IL-10, IFN-γ, and IL-17A were the eight independent variables that were filtered out when the mean square error was at its lowest (Supplementary Figure S3b). The value of λ was 0.01175831. The three independent variables that were screened were age group, IL-6, and IL-10, and the value of λ was 0.07905534 when the distance from the mean square error was one standard error (Supplementary Figure S3b). Meanwhile, age group was found to have interactions with IL-5 and IL-10 (Supplementary Figure S4a,b), so interactive variables were added. Therefore, in the multivariate logistic regression analysis, age group, IL-5, IL-6, IL-8, IL-10, IFN-γ, IL-17A, age-group*IL-5, and age-group*IL-10 were included. The final model included: Age group (*p* = 0.0062), IL-6 (*p* = 0.0015), IL-17A (*p* = 0.0474), age-group*IL-5 (*p* = 0.0091), and age-group*IL-10 (*p* = 0.0209) (Table 5).

**Figure 2:**
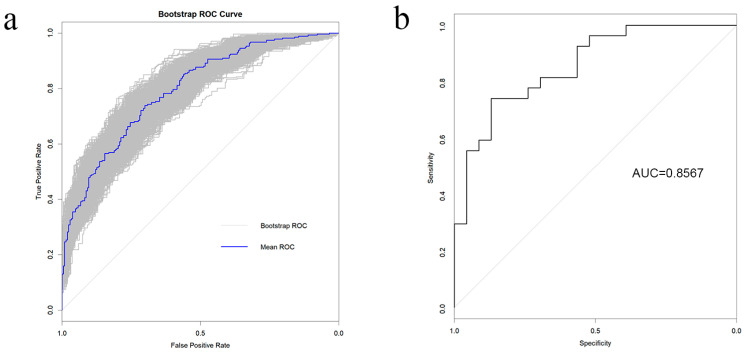
Model performance. (**a**) ROC curve of 1000-time bootstrap; (**b**) ROC curve of external test.

In this model, the CA clinical prediction model equation was *p* = 1/[1 + exp(−0.7197 age-group + 0.0139 IL-6 + 0.0475 IL-17A − 1.6430 age-group*IL-5 + 0.1469 age-group*IL-10)], with a model cutoff value of 0.5114658, an AUC of 0.7949 (95% CI 0.7558–0.834), and prediction sensitivity and specificity of 70.0% and 73.9%, respectively. Meanwhile, the positive predictive value for CA was 72.9% and the negative predictive value was 71.1%. Subsequently, the model underwent both internal and external validation. For internal validation, a random sample of 1000 repeats was chosen, and the bootstrap results revealed that the prediction model’s mean AUC was 0.792 (Figure 2a). Plotting the calibration performance (Supplementary Figure S3c) and clinical decision curve analysis (DCA) (Supplementary Figure S3d) revealed that the model had good accuracy between the true value and the calibration value and that the intervention of CA based on the current prediction model could have a high benefit.

To validate the current model, an additional 50 AA children from the Children’s Hospital of Chongqing Medical University were recruited as an external cohort. The Hosmer-Lemeshow goodness-of-fit test was run, and the results revealed *p* = 0.06949, indicating that the model was well-calibrated for external validation data testing. Plotting the ROC curve to evaluate the model’s discrimination (Figure 2b) revealed an AUC of 0.8567, indicating a high degree of model accuracy.

## 4. Discussion

Cytokines, small protein molecules synthesized by both immune and non-immune cells, have a significant impact on human inflammation and various disease processes. When the body is infected or injured, cytokines trigger inflammatory responses, making them valuable as biomarkers for the diagnosis of inflammation-related diseases, reflecting the status of the immune system and inflammatory responses. Appendicitis is the most common abdominal surgical emergency in children, and early identification of its type is crucial for determining the appropriate treatment and improving prognosis in children with AA [13]. The inflammatory process of appendicitis involves a complex interplay of various cytokines, which have been implicated in the progression and severity of the disease. Elevated levels of pro-inflammatory cytokines may suggest a more aggressive inflammatory response. Conversely, anti-inflammatory cytokines may play a protective role by dampening the inflammatory cascade and limiting tissue damage. By measuring the levels of specific cytokines in blood or tissue samples, clinicians may be able to differentiate between uncomplicated and complicated appendicitis, thereby guiding treatment decisions. Cytokines may also serve as prognostic indicators in complicated appendicitis. By monitoring the levels of specific cytokines over time, clinicians can assess the response to treatment and predict the risk of complications such as abscess formation or peritoneal contamination.

The current study, encompassing 483 pediatric patients diagnosed with acute appendicitis, represents the first investigation to comprehensively evaluate twelve cytokines and their potential in discriminating between uncomplicated and complicated cases of acute appendicitis in children, as well as their prognosis prediction abilities. In this study, children with acute appendicitis were divided into a complicated appendicitis group (CA) and an uncomplicated appendicitis group (UA). We discovered that the risk of CA increased with advancing age among children. An extended duration of preoperative abdominal pain along with fever and symptoms of vomiting was associated with a higher probability of CA. Furthermore, children in the CA group exhibited a poorer prognosis, characterized by prolonged hospital stays and an increased requirement for carbapenem antibiotics. They also had a heightened risk of complications, such as sepsis, intestinal adhesions, and intestinal obstruction, consistent with previous research [14,15]. In particular, higher IL-6, IL-8, and IL-10 levels were associated with a greater need for carbapenem antibiotics, postoperative admission to PICU, and complications. We then carried out an analysis to determine the connection between cytokines and children’s AA stage and prognosis. We discovered that preoperative serum levels of IFN-γ, IL-5, IL-6, IL-8, and IL-10 hold diagnostic value in staging pediatric acute appendicitis. Among them, IL-6, IL-8, and IL-10 demonstrated superior predictive capabilities for CA in children, with AUC values of 0.794, 0.604, and 0.746 at cutoff values of 32.135 pg/mL, 7.750 pg/mL, and 4.395 pg/mL, respectively.

A great number of studies have already confirmed the precise involvement of IL-6 and IL-8 in appendicitis staging. During the onset of inflammation, IL-6 surges, triggering the synthesis of acute chronophilin while suppressing the production of fibronectin, albumin, and transferrin. It has been demonstrated that there is a positive correlation between serum IL-6 expression and an increase in the inflammatory response [16,17], which may effectively respond to the level of inflammation within the body [18] and plays a crucial role in determining infection prognosis and severity [19]. Yoon et al. [20] and others [21] reported higher preoperative blood levels of IL-6 in children with appendiceal perforations compared to non-perforated children. Other studies have demonstrated that serum IL-6 can differentiate between complicated and uncomplicated appendicitis. The findings of our study align with these previous investigations, as we observed that among the twelve cytokines, when used individually for CA prediction, IL-6 exhibited the highest predictive value with a specificity of up to 81.6%.

IL-8 is primarily produced by activated monocytes, immune cells, and epithelial cells, which plays a significant role in triggering inflammatory responses and tissue damage by attracting and activating neutrophils, causing neutrophil influx into tissues, activating neutrophils, and degranulating their intracellular stores, therefore fostering an inflammatory reaction. Histologic scores and IL-8 expression levels exhibited a robust association [22]. Additionally, immunohistochemistry revealed the presence of IL-8 protein in both neutrophils and monocytes, indicating its potential involvement in the pathogenesis of appendicitis. Children with simple appendicitis, suppurative appendicitis, and gangrenous appendicitis displayed a progressive rise in serum IL-8 levels, according to Mo et al. [23]. Similarly, Rubér discovered a significantly elevated level of IL-8 in cases of perforated appendicitis [24]. We also discovered significant differences in IL-8 levels between children with CA and UA, although its predictive value for CA was limited to a specificity of only 55.1%.

TNF-α is crucial for regulating immune responses, eliminating target cells, and triggering apoptosis, which exerts a protective effect on the mucosa during acute injury and promotes inflammation in chronic inflammatory conditions [25]. As the primary mediator of the inflammatory response in living organisms, TNF-α stimulates T cells to release additional inflammatory mediators and cytokines. This, in turn, triggers a cascade of inflammatory reactions in the body, contributing to the progression of diseases. Previous studies have demonstrated the positive association of TNF-α with complicated appendicitis, distinguishing it from uncomplicated cases [26,27]. Children with complicated acute appendicitis had considerably higher serum TNF-α levels compared to those with uncomplicated appendicitis, according to two investigations conducted on children with AA [28,29]. Nevertheless, our study did not observe any discernible difference in preoperative serum TNF-α levels between children with CA and UA.

The cytokine IL-10, which has immunosuppressive and immunomodulatory properties, is produced by T cells, monocytes, B cells, and macrophages. Previous research has yielded conflicting results. Some investigations have discovered a correlation between IL-10 and the severity of appendicitis [26]. Serum levels of IL-10 were significantly elevated in children with perforated appendicitis [30,31]. However, Dadal et al.’s study [32] did not discover any appreciable variation in serum levels of IFN-γ and IL-10 between children with or without appendicitis. IL-10 levels were observed to be lower in patients with appendicitis by de Oliveira et al. [33]. The results of our study demonstrated the utility of both IFN-γ and IL-10 levels in identifying CA in children. When used as an independent predictor, IL-10 exhibited good predictive efficacy with a sensitivity of 64.1%.

Limited previous studies have been conducted on other cytokines. Groselj et al.’s study [21] discovered that children with appendiceal perforations had higher serum IL-12 levels. However, we did not find IL-12 to be a useful marker for distinguishing between CA and UA in children. Carvalho et al. [34] reported no significant influence of serum IL-4 levels on the risk of complicated appendicitis, which is consistent with our findings. A study investigating seventeen different cytokines in patients with appendicitis showed no statistically significant changes in preoperative levels of cytokines such as IFN-α and IL-1β [35]. Nonetheless, IL-1β was identified by Rubér et al. as a valuable diagnostic indicator in foretelling acute appendicitis [36]. However, we found no significant difference in IL-1β levels between CA and UA. Another investigation on IL-5 discovered a favorable correlation between blood and peritoneal lavage fluid levels of IL-5 and the severity of appendiceal inflammation [37]. Similar results were found in our study, which showed that children with CA had considerably greater preoperative serum IL-5 levels than those with UA.

We investigated the risk factors for CA in comparison to UA to build a useful model for predicting CA. Through multifactorial logistic analysis, we identified age, IL-10, and IFN-γ as independent risk factors for CA. Notably, IFN-γ and age exhibited a protective effect among these variables. The combination of age, IL-10, and IFN-γ yielded an AUC of 0.7949 in our CA prediction model, demonstrating a predictive sensitivity of 70.0% and specificity of 73.9%. To mitigate bias and prevent overfitting issues, we employed Bootstrap for internal validation while also conducting external validation experiments. These validations consistently confirmed the stability and high efficacy of our developed model. Importantly, all factors included in the model are objective measures that minimize potential biases arising from parental or child recollections. Consequently, we propose that cytokine testing can serve as a valuable tool for predicting CA in children with AA before surgery by providing clinicians with reliable reference values to facilitate early therapeutic decision-making.

Although the use of cytokines such as IL-6, IL-8, and IL-10 for CA prediction has been extensively documented, there is a lack of large-sample studies investigating various cytokines. The advantages of our study include substantial sample size, comprehensive analysis encompassing a wide range of cytokines, incorporation of twelve cytokines in variable screening, and development of a clinical prediction model for CA. However, this study also has certain limitations. Firstly, it is a retrospective study that only included individuals who underwent appendectomy while excluding data from clinically suspected cases without surgery. Additionally, being conducted at a single center may have resulted in the incomplete acquisition of medical data about all risk variables associated with pediatric CA, potentially leading to biased results. To further validate the findings of this study, greater sample sizes and various centers in randomized controlled trials are required in future research. Furthermore, our model also has limitations in cases of suspected uncomplicated appendicitis. Determining whether such patients should undergo conservative treatment or aggressive surgery poses a dilemma. We recommend that in such situations, a comprehensive judgment should be made by considering other clinical information, including the patient’s clinical manifestations, imaging examination results, laboratory test results, as well as the outcomes of our clinical prediction model. According to current studies, conservative strategies as the initial treatment for pediatric patients with uncomplicated appendicitis may be feasible and effective without increasing the risk for complications [38]. Regardless, we believe that in actual clinical practice, pediatric surgeons need to consider multiple factors comprehensively to make the best clinical treatment decisions.

In summary, the accurate classification and prognostic prediction of AA in children are increasingly crucial in the current clinical setting. Therefore, it is critical to investigate factors that contribute to a better understanding of pediatric AA classification and prognosis, enabling prompt recognition of CA by clinicians for early clinical decision-making. While individual cytokines such as IL-6, IL-8, and IL-10 demonstrated moderate effectiveness in distinguishing between pediatric CA and UA, a multifactorial logistic regression prediction model incorporating age, IL-10, and IFN-γ exhibited good predictive efficacy for identifying CA. In conclusion, incorporating cytokine levels into the assessment of pediatric acute appendicitis can potentially enhance clinical outcomes in children with AA by anticipating the occurrence of CA and improving their prognosis.

## Figures and Tables

**Table 1:** Baseline information and clinical characteristics.

Variables	UA (*n* = 207)	CA (*n* = 276)	Z-Value	*p*-Value
Age (y), median (IQR)	9.6 (5.20)	8.1 (4.50)	−3.763	<0.001
Younger age group (y), median (IQR)	6.8 (2.88)	6.9 (2.80)		
Elder age group (y), median (IQR)	12.1 (2.33)	11.4 (2.35)		
Sex (M/total), *n* (%)	124 (59.9)	168 (60.9)	0.046	0.83
Hours of pain evolution (h), median (IQR)	20 (39)	48 (48)	−6.859	<0.001
Fever (yes/total), *n* (%)	40 (19.3)	114 (41.3)	26.314	<0.001
Vomiting (yes/total), *n* (%)	90 (43.5)	202 (73.2)	43.674	<0.001
Diarrhea (yes/total), *n* (%)	39 (18.8)	65 (23.6)	1.553	0.213

UA: uncomplicated appendicitis, CA: complicated appendicitis, IQR: interquartile range.

**Table 2:** Cytokines levels.

Cytokines (pg/mL)	UA (*n* = 207)	CA (*n* = 276)	Z-Value	*p*-Value	Cut-Off	Sensitivity (%)	Specificity (%)
Median (IQR)							
IL-1β	1.65 (2.38)	1.92 (2.46)	−1.645	0.100			
IL-2	0.59 (0.87)	0.59 (0.91)	−1.128	0.259			
IL-4	0.78 (1.35)	0.73 (1.40)	−1.134	0.257			
IL-5	0.47 (0.62)	0.31 (0.61)	−2.799	0.005			
IL-6	11.69 (24.50)	55.86 (250.81)	−11.091	<0.001	32.135	62.7	81.6
IL-8	6.96 (11.13)	10.33 (18.95)	−4.025	<0.001	7.75	63.8	55.1
IL-10	2.72 (2.97)	6.48 (20.17)	−9.293	<0.001	4.395	64.1	73.4
IL-12	1.24 (1.77)	1.05 (1.78)	−1.620	0.105			
IL-17A	3.44 (4.53)	3.46 (5.95)	−0.379	0.705			
TNF-α	0.99 (1.18)	1.00 (1.48)	−0.332	0.740			
IFN-α	1.45 (4.27)	1.88 (5.55)	−1.486	0.137			
IFN-γ	0.87 (1.50)	0.67 (0.79)	−3.733	<0.001			

UA: uncomplicated appendicitis, CA: complicated appendicitis, IQR: interquartile range, IL-1β: interleukin-1β, IL-2: interleukin-2, IL-4: interleukin-4, IL-5: interleukin-5, IL-6: interleukin-6, IL-8: interleukin-8, IL-10: interleukin-10, IL-12: interleukin-12, IL-17A: interleukin-17A, TNF-α: tumor necrosis factor-α, IFN-α: interferon-α, IFN-γ: interferon-γ, IQR: interquartile range.

**Table 3:** Prognostic indicators.

Variables	UA (*n* = 207)	CA (*n* = 276)	Z-Value	*p*-Value
Hospital stay (d), median (IQR)	7 (6, 8)	9 (8, 11)	−12.493	<0.001
Carbapenems antibiotics (yes/total), (*n*%)	1 (0.5)	32 (11.6)	22.941	<0.001
PICU (yes/total), (*n*%)	2 (1)	5 (1.8)	0.592	0.442
Complications (yes/total), (*n*%)	40 (19.3)	257 (93.1)	272.006	<0.001

UA: uncomplicated appendicitis, CA: complicated appendicitis, IQR: interquartile range.

**Table 4:** Cytokines and prognosis.

Variables	Carbapenems Antibiotics			PICU			Complications		
	Y (*n* = 33)	N (*n* = 450)	*p*-Value	Y (*n* = 7)	N (*n* = 476)	*p*-Value	Y (*n* = 298)	N (*n* = 185)	*p*-Value
IL-1β	2.47 (3.88)	1.77 (2.44)	<0.001	1.97 (2.10)	1.81 (2.47)	0.965	1.96 (2.48)	1.46 (2.25)	0.009
IL-2	0.80 (1.09)	0.59 (0.90)	0.469	0.90 (0.66)	0.59 (0.91)	0.525	0.59 (0.93)	0.59 (0.82)	0.597
IL-4	0.73 (1.56)	0.74 (1.39)	0.875	0.02 (0.93)	0.75 (1.41)	0.188	0.72 (1.40)	0.78 (1.38)	0.414
IL-5	0.30 (1.27)	0.39 (0.60)	0.382	0.12 (3.00)	0.38 (0.62)	0.443	0.35 (0.67)	0.45 (0.55)	0.203
IL-6	118.81 (602.92)	25.18 (58.68)	<0.001	193.31 (835.36)	26.44 (66.93)	0.042	50.42 (210.60)	12.97 (25.39)	<0.001
IL-8	35.14 (64.19)	8.23 (13.25)	<0.001	37.89 (102.54)	8.65 (15.52)	0.011	10.20 (19.87)	7.22 (10.15)	<0.001
IL-10	37.62 (68.07)	3.92 (6.71)	<0.001	80.99 (143.63)	4.09 (7.67)	0.021	5.98 (19.43)	2.62 (3.23)	<0.001
IL-12	0.97 (1.94)	1.13 (1.69)	0.185	0.42 (1.04)	1.12 (1.70)	0.081	1.08 (1.75)	1.23 (1.66)	0.268
IL-17A	2.81 (6.12)	3.47 (5.38)	0.402	5.12 (10.96)	3.44 (5.33)	0.644	3.50 (5.71)	24.94 (4.58)	0.820
TNF-α	0.90 (1.74)	1.00 (1.40)	0.806	0.88 (1.86)	0.99 (1.40)	0.631	1.06 (1.55)	0.98 (1.12)	0.228
IFN-α	3.09 (10.98)	1.59 (4.58)	0.060	1.73 (17.60)	1.68 (4.93)	0.620	2.05 (6.22)	1.30 (3.17)	0.005
IFN-γ	0.64 (0.84)	0.75 (1.01)	0.719	0.64 (1.60)	0.72 (1.00)	0.696	0.68 (0.80)	0.87 (1.41)	0.007

UA: uncomplicated appendicitis, CA: complicated appendicitis, IQR: interquartile range, IL-1β: interleukin-1β, IL-2: interleukin-2, IL-4: interleukin-4, IL-5: interleukin-5, IL-6: interleukin-6, IL-8: interleukin-8, IL-10: interleukin-10, IL-12: interleukin-12, IL-17A: interleukin-17A, TNF-α: tumor necrosis factor-α, IFN-α: interferon-α, IFN-γ: interferon-γ, Y: yes, N: no, IQR: interquartile range.

**Table 5:** Multivariate logistic regression analysis of risk factors for CA in pediatric AA.

Variables	β-Value	OR	95%CI		*p*-Value
			Lower Limit	Upper Limit	
Intercept	0.3726				0.2929
Age group	−0.0808	0.6730000	0.4871700	0.929710	0.0163
IL-5	0.0044	0.0026846	−0.0057392	0.011108	0.5322
IL-6	−0.0001	0.9933200	0.9287100	1.062400	0.8452
IL-8	−0.0020	0.9683400	0.8649700	1.084100	0.5764
IL-10	0.1069	2.4679000	1.7362000	3.508000	<0.0001
IL-17A	0.0294	1.1711000	0.9445800	1.452000	0.1498
IFN-γ	−0.2161	0.8056300	0.7153100	0.907360	0.0004

OR: odds ratio, CI: confidence interval, IL-5: interleukin-5, IL-6: interleukin-6, IL-8: interleukin-8, IL-10: interleukin-10, IL-17A: interleukin-17A, IFN-γ: interferon-γ.

## Data Availability

The datasets generated and analyzed during the current study are available from the corresponding author upon reasonable request due to ethical reasons.

## Supplementary Materials

The following supporting information can be downloaded at: https://www.mdpi.com/article/10.3390/children12030298/s1, Figure S1: PCA and correlation analysis between cytokines; Figure S2: ROC curves of cytokines in distinguish CA and UA; Figure S3: Lasso regression curve and internal tsets; Figure S4: Interactions between IL-5, IL-10 and age-group; Table S1: *p*-Values of interactions between patient age, sex, and cytokine levels.
