# Supplementary material for: Discriminatory Value and Validation of a Risk Prediction Model Based on Serum Cytokines in Pediatric Acute Appendicitis: A Single-Center Experience of 483 Cases
Source: Children (Basel). 2025 Feb 27;12(3):298. doi: 10.3390/children12030298 (PMC11941304; doi:10.3390/children12030298)
Supplement: Supplementary file 1 [file children-12-00298-s001.zip › Supplementary Table S1.pdf]

Supplementary Table S1. P-values of interactions between patient age, sex, and cytokine levels.

Cytokines (pg/mL)	Age	Sex
IL-1β	<0.001	0.838
IL-2	0.012	0.827
IL-4	0.207	0.838
IL-5	0.010	0.357
IL-6	<0.001	0.497
IL-8	<0.001	0.313
IL-10	<0.001	0.300
IL-12	0.102	0.898
IL-17A	0.106	0.942
TNF-α	0.036	0.185
IFN-α	<0.001	0.629
IFN-γ	0.541	0.669
