# Supplementary figures and images for: Discriminatory Value and Validation of a Risk Prediction Model Based on Serum Cytokines in Pediatric Acute Appendicitis: A Single-Center Experience of 483 Cases
Source: Children (Basel). 2025 Feb 27;12(3):298. doi: 10.3390/children12030298 (PMC11941304; doi:10.3390/children12030298)

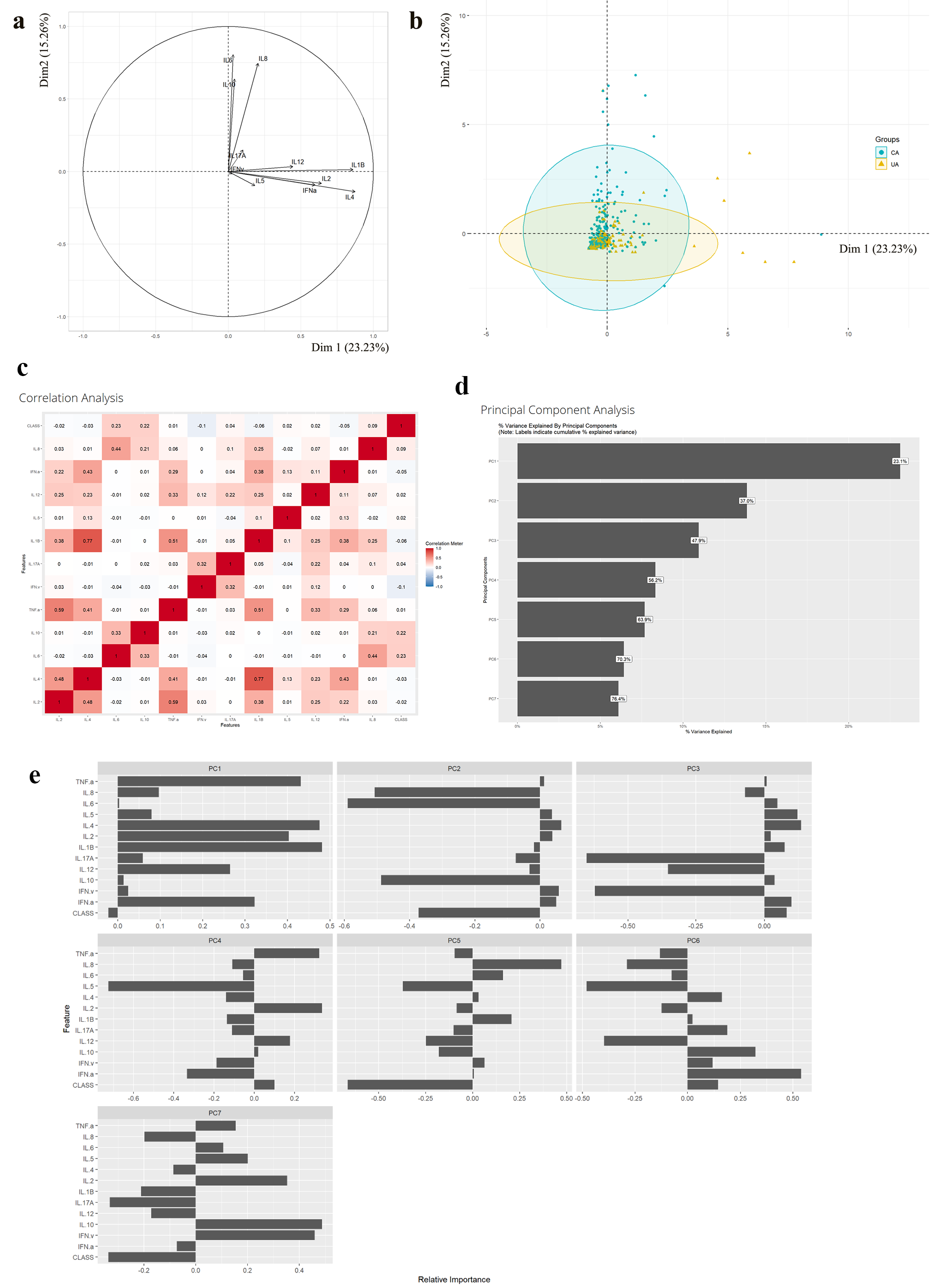

Supplement: Supplementary file 1 [file children-12-00298-s001.zip › Supplementary Figure S1.tif]

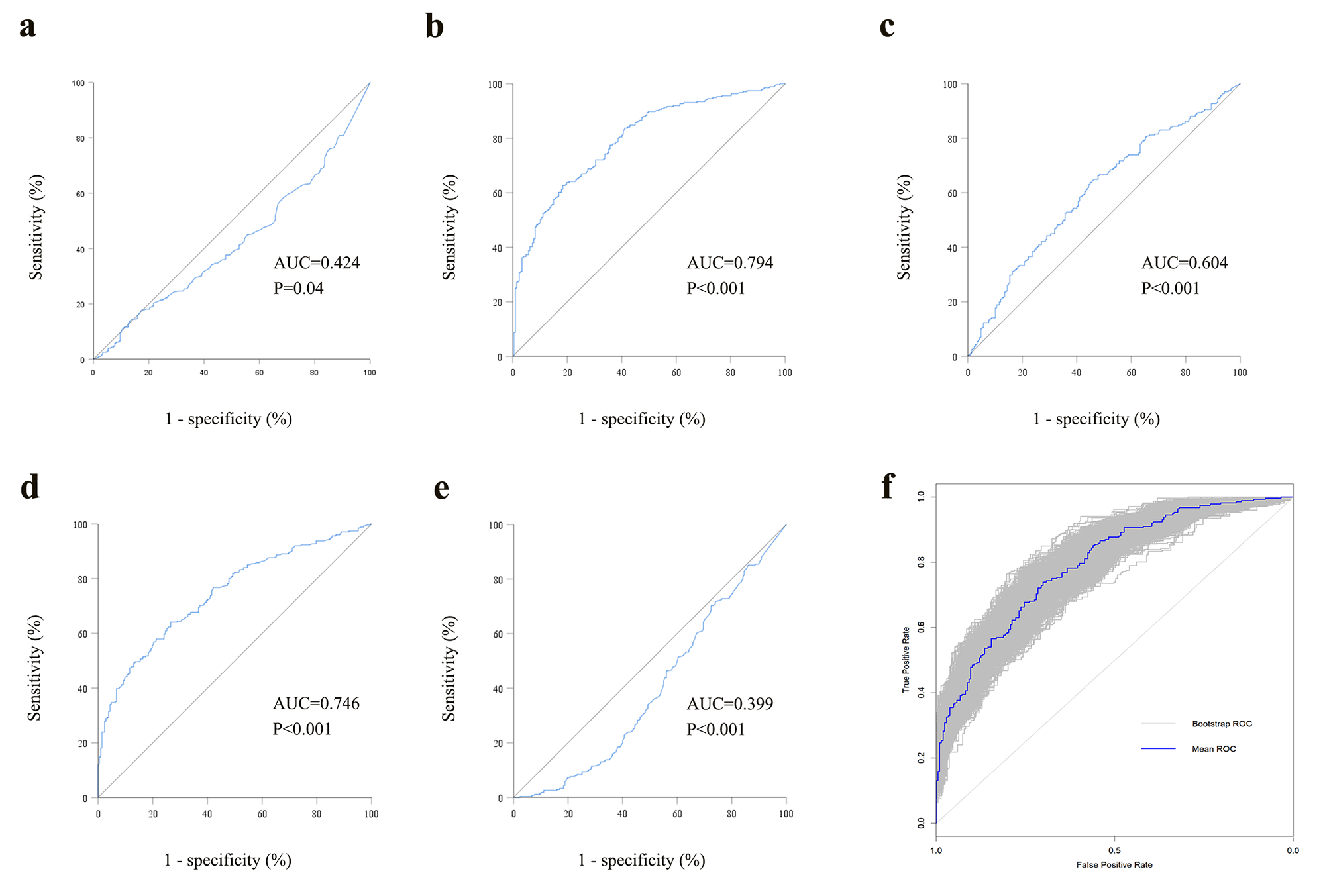

Supplement: Supplementary file 1 [file children-12-00298-s001.zip › Supplementary Figure S2.tif]

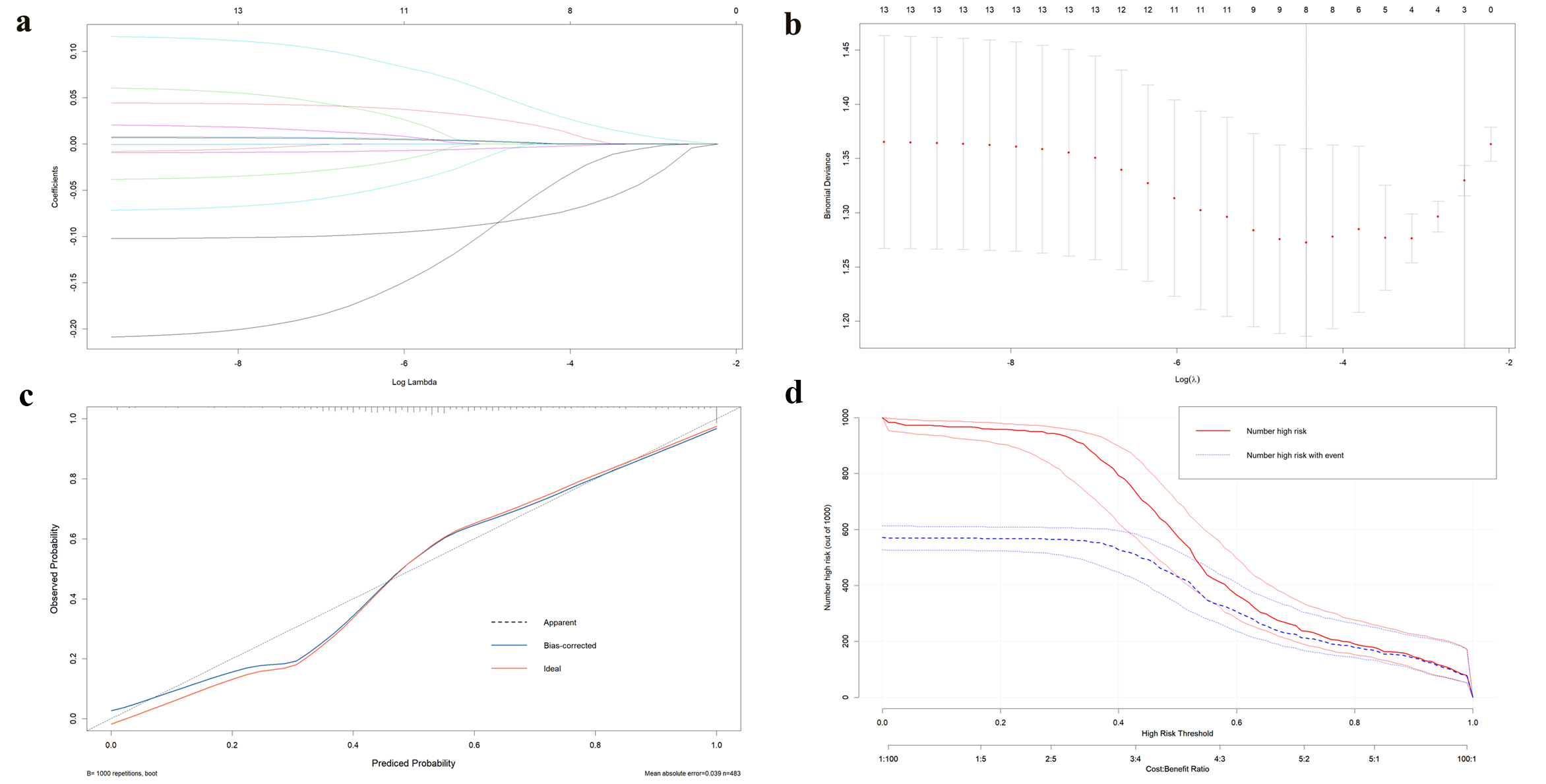

Supplement: Supplementary file 1 [file children-12-00298-s001.zip › Supplementary Figure S3.tif]

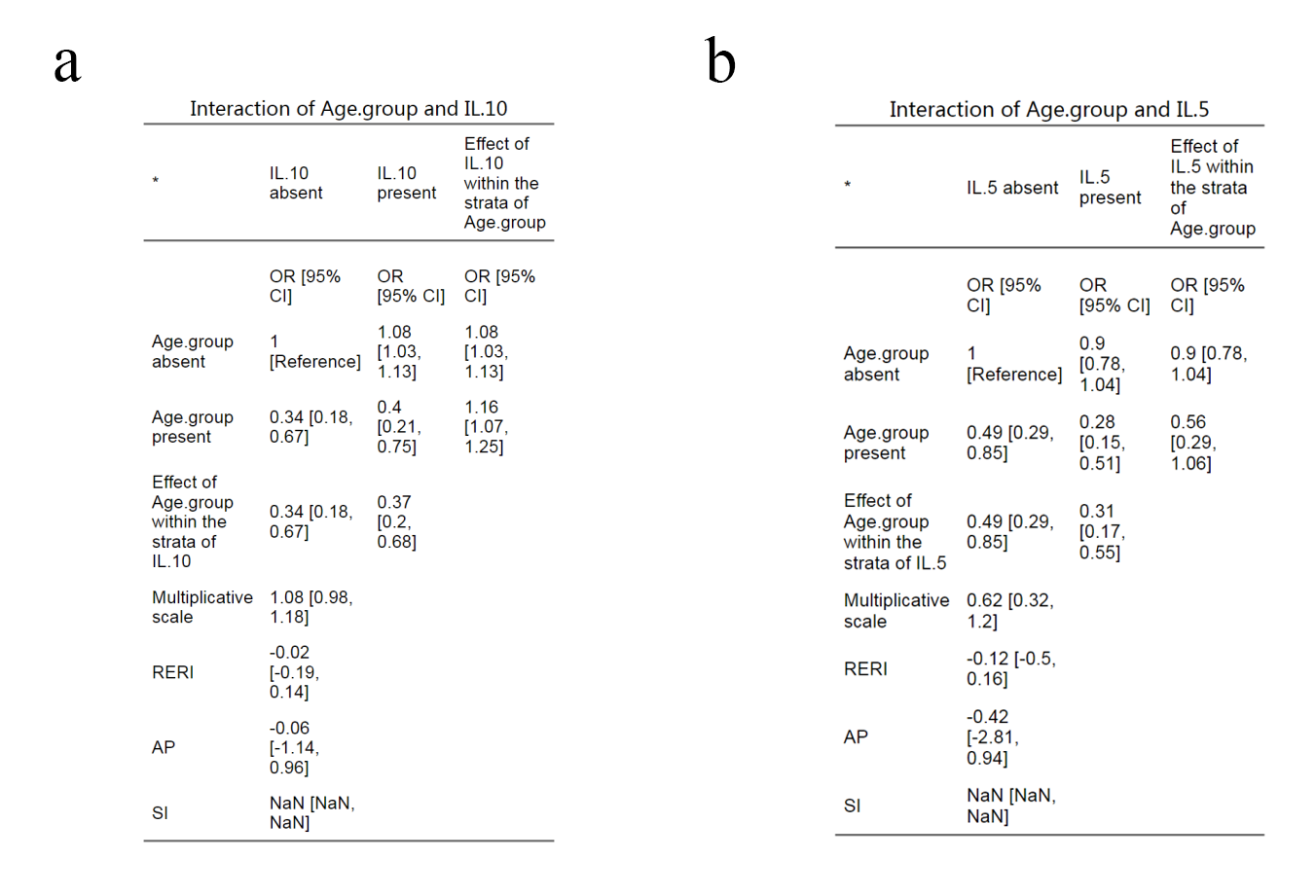

Supplement: Supplementary file 1 [file children-12-00298-s001.zip › Supplementary Figure S4.tif]
